# Distinct Infection Mechanisms of *Rhizoctonia solani* AG-1 IA and AG-4 HG-I+II in *Brachypodium distachyon* and Barley

**DOI:** 10.3390/life15020235

**Published:** 2025-02-05

**Authors:** Niranjan Mahadevan, Rozi Fernanda, Yusuke Kouzai, Natsuka Kohno, Reiko Nagao, Khin Thida Nyein, Megumi Watanabe, Nanami Sakata, Hidenori Matsui, Kazuhiro Toyoda, Yuki Ichinose, Keiichi Mochida, Hiroshi Hisano, Yoshiteru Noutoshi

**Affiliations:** 1 Graduate School of Environmental, Life, Natural Science and Technology, Okayama University, Okayama 700-8530, Japan; madava06@gmail.com (N.M.); rozifernanda86@gmail.com (R.F.); nanami.sakata@okayama-u.ac.jp (N.S.); hmatsui@okayama-u.ac.jp (H.M.); pisatin@okayama-u.ac.jp (K.T.); yuki@okayama-u.ac.jp (Y.I.); 2 Tea Research Institute of Sri Lanka, St. Coombs, Talawakelle 22100, Sri Lanka; 3 Crop Stress Management Group, Division of Plant Molecular Regulation Research, Institute of Agrobiological Sciences, National Agriculture and Food Research Organization (NARO), Tsukuba 305-8634, Japan; kozaiy750@affrc.go.jp; 4 Faculty of Agriculture, Okayama University, Okayama 700-8530, Japan; 5 RIKEN Center for Sustainable Resource Science, Yokohama 230-0045, Japan; keiichi.mochida@riken.jp; 6 Kihara Institute for Biological Research, Yokohama City University, Yokohama 244-0813, Japan; 7 School of Information and Data Sciences, Nagasaki University, Nagasaki 852-8521, Japan; 8 Institute of Plant Science and Resources, Okayama University, Kurashiki 710-0046, Japan; hiroshi.hisano@rib.okayama-u.ac.jp

**Keywords:** *Rhizoctonia solani* species complex, virulence mechanism, infection behavior, salicylic acid, *N*-hydroxypipecolic acid

## Abstract

*Rhizoctonia solani* is a basidiomycete phytopathogenic fungus that causes rapid necrosis in a wide range of crop species, leading to substantial agricultural losses worldwide. The species complex is divided into 13 anastomosis groups (AGs) based on hyphal fusion compatibility and further subdivided by culture morphology. While *R. solani* classifications were shown to be independent of host specificity, it remains unclear whether different *R. solani* isolates share similar virulence mechanisms. Here, we investigated the infectivity of Japanese *R. solani* isolates on *Brachypodium distachyon* and barley. Two isolates, AG-1 IA (from rice) and AG-4 HG-I+II (from cauliflower), infected leaves of both plants, but only AG-4 HG-I+II infected roots. *B. distachyon* accessions Bd3-1 and Gaz-4 and barley cultivar ‘Morex’ exhibited enhanced resistance to both isolates compared to *B. distachyon* Bd21 and barley cultivars ‘Haruna Nijo’ and ‘Golden Promise’. During AG-1 IA infection, but not AG-4 HG-I+II infection, resistant Bd3-1 and Morex induced genes for salicylic acid (SA) and *N*-hydroxypipecolic acid (NHP) biosynthesis. Pretreatment with SA or NHP conferred resistance to AG-1 IA, but not AG-4 HG-I+II, in susceptible *B. distachyon* Bd21 and barley Haruna Nijo. On the leaves of susceptible Bd21 and Haruna Nijo, AG-1 IA developed extensive mycelial networks with numerous infection cushions, which are specialized infection structures well-characterized in rice sheath blight. In contrast, AG-4 HG-I+II formed dispersed mycelial masses associated with underlying necrosis. We propose that the *R. solani* species complex encompasses at least two distinct infection strategies: AG-1 IA exhibits a hemibiotrophic lifestyle, while AG-4 HG-I+II follows a predominantly necrotrophic strategy.

## 1. Introduction

*Rhizoctonia solani* Khün, a soilborne necrotrophic basidiomycete fungus, is one of the most destructive plant pathogens due to its wide host range and geographical distribution [1]. The pathogen infects over 200 plant species worldwide, including economically important crops and ornamental plants [2], with 188 host species from 32 families reported in Japan alone [3]. On rice leaves, *R. solani* forms specialized structures, called infection cushions, when mycelia or sclerotia contact the host surface. These structures facilitate host colonization by degrading plant cell walls, ultimately leading to tissue death. In addition to infection cushions, lobate appressoria and direct mycelial penetration through stomata have also been observed [4]. During the infection, *R. solani* isolates release various types of carbohydrate-active enzymes (CAZymes) to degrade plant tissue, as well as small secreted proteins called effectors, which possess necrosis-inducing or immunity-suppressing activities [5,6,7,8]. Following infection, the fungus produces resilient sclerotia, which can persist in soil for years, ensuring long-term survival [9,10]. Symptoms of *R. solani* infection include foliar and sheath blights, damping-off, crown rot, stem rot, root rot, and seed decay [11].

*R. solani* isolates are classified into 13 anastomosis groups (AG-1 through AG-13) based on hyphal fusion reactions [12]. These AGs are further subdivided according to DNA sequence features, colony morphology, host range, pathogenicity, and nutritional requirements, establishing *R. solani* as a species complex [13]. In Japan, eight AGs (AG-1 through AG-7 and AG-11) are naturally present in agroecosystems [14]. However, AG classification does not strictly correlate with host or tissue specificity. For example, three AG-1 IA isolates from different hosts (two from rice and one from sudangrass) all infected the leaves of the monocot model plant *Brachypodium distachyon* [15]. Yet, when tested on the dicot model *Arabidopsis thaliana*, only one isolate infected both roots and leaves, while the other two were non-virulent [16]. Similarly, among three AG-2-2 IIIB isolates from rice, broccoli, and Welch onion, all infected Arabidopsis roots, but only one infected its leaves [16]. These findings indicate that virulence and host specificity in *R. solani* likely evolved independently of AG classification, although the conservation of infection mechanisms among isolates remains poorly understood.

Plant defense mechanisms against fungal pathogens consist of preformed and induced responses. Preformed defenses include physical barriers, such as the cuticle and cell walls, as well as chemical barriers, including phytoanticipins and inhibitory proteins. To successfully invade and obtain nutrients, pathogens must overcome these initial defenses. During invasion, host enzymes degrade a pathogen’s cell walls, releasing microbe-associated molecular patterns (MAMPs). The recognition of MAMPs triggers pattern-triggered immunity (PTI), which is characterized by the formation of papillae and the synthesis of phytoalexins. To establish biotrophic interactions, some pathogens suppress PTI by deploying effector proteins. Plants counteract this suppression through nucleotide-binding leucine-rich repeat (NLR) receptors, which recognize these effectors and activate effector-triggered immunity (ETI), a more robust and specific form of PTI [17]. This recognition also induces systemic acquired resistance (SAR), which primes uninfected tissues for enhanced defense against future infections. In contrast, necrotrophic pathogens kill host cells using CAZymes and toxins. Application of hydraulic force, ice nucleation, induction of reactive oxygen species production, creation of an acidic environment, manipulation of the regulated cell death machinery, etc., also function as virulence mechanisms [18,19,20,21]. However, pathogen infection strategies are not strictly dichotomous [22]. Some necrotrophic pathogens including *R. solani* employ effectors to enhance virulence, like biotrophic and hemibiotrophic pathogens, as described above.

Phytohormones are critical regulators of plant immunity. Salicylic acid (SA) activates defenses against biotrophic and hemibiotrophic pathogens, while jasmonic acid (JA) and/or ethylene (ET) mediate responses to necrotrophic pathogens [23,24,25,26]. Among them, SA is biosynthesized in plants through two major pathways: the isochorismate synthase (ICS) pathway and the phenylalanine ammonia-lyase (PAL) pathway. In the ICS pathway of Arabidopsis, chorismate is converted to isochorismate by ICS. Isochorismate is then transported to the cytoplasm by Enhanced Disease Susceptibility 5 (EDS5). AvrPphB susceptible 3 (PBS3) conjugates isochorismate to glutamate (Glu) to produce IC-9-Glu. Enhanced Pseudomonas Susceptibility 1 (EPS1) cleaves IC-9-Glu to SA [27]. Recently, *N*-hydroxy pipecolic acid (NHP) has been identified as a key signaling molecule in SAR [28,29,30]. NHP biosynthesis at infection sites follows a three-step pathway: AGD2-LIKE DEFENSE RESPONSE PROTEIN 1 (ALD1) converts L-lysine to dehydropipecolic acid, SAR DEFICIENT 4 (SARD4) reduces it to pipecolic acid (Pip), and FLAVIN-DEPENDENT MONOOXYGENASE 1 (FMO1) converts Pip to NHP [30,31]. Both NHP and Pip confer resistance in dicots and monocots, showing strong efficacy against biotrophic and hemibiotrophic pathogens, but limited activity against necrotrophic pathogens [29,31,32].

Phytohormone-mediated defense responses against *R. solani* vary depending on the infection site, pathogen isolate, and host species. For instance, in *A. thaliana*, single mutations in SA, ET, JA, ABA, camalexin, or auxin biosynthesis or signaling pathways did not affect root infection by *R. solani* AG-2-1 and AG-8 [33]. However, combined mutations in ET (*ein2*), JA (*coi1*), and PENETRATION2 (*pen2*) pathways compromised the defense against AG-8 in both the leaves and roots [34]. In *Medicago truncatula,* ET signaling was essential for flavonoid-mediated resistance to AG-8 root infection [35]. Our previous studies demonstrated that exogenous SA application induced resistance to AG-1 IA in *B. distachyon* and rice, but not in *A. thaliana* [15,16]. Furthermore, rice plants expressing the bacterial SA hydrolase gene (*NahG*) showed increased susceptibility to *R. solani* [15]. In tomato, SA priming provided protection against AG-1 IA compared to JA priming [36]. Conversely, JA application induced resistance to AG-1 IA in rice [37], while in Tartary buckwheat, JA was the primary mediator of resistance against AG-4 HGI3 [38]. These findings suggest that different *R. solani* isolates employ distinct infection strategies that are countered by either SA- or JA-mediated defenses independently of their AGs classification. Notably, the potential effects of exogenous NHP application on plant resistance to *R. solani* remain unexplored.

In this study, we compared the virulence of two Japanese *R. solani* isolates, AG-1 IA and AG-4 HG-I+II, using *B. distachyon* and barley (*Hordeum vulgare*) as host plants. Despite belonging to a single species complex, these isolates exhibited different infection mechanisms. Our results challenge the long-held assumption of uniform pathogenicity strategies within *R. solani*. Our findings provide new insights into the biology and ecology of this fungal species, advancing its taxonomic classification and our understanding of its pathogenic behavior. This knowledge may inform the development of more effective, pathotype-specific strategies for managing *R. solani* and reducing its impact on global agriculture.

## 2. Materials and Methods

### 2.1. Fungal and Plant Materials

Eight *R. solani* isolates representing six anastomosis groups were obtained from the Genebank of the National Agricultural Research Organization (NARO), Tsukuba, Japan. The isolates were maintained on potato dextrose agar (PDA) medium (24 g/L Difco^™^ potato dextrose broth, 20 g/L Bacto^™^ agar) at 23 °C for 3–5 days before inoculation experiments. Stock cultures were preserved on PDA slants at 4 °C.

Seeds of *B. distachyon* and barley were obtained from the National Plant Germplasm System of the United States Department of Agriculture–Agriculture Research Service (USDA-ARS) and the National Bioresource Project (NBRP) in charge at the Institute of Plant Sciences and Resources (IPSR), Kurashiki, Okayama University, Japan, respectively. The seeds were stored in a Dry keeper^™^ cabinet at 25 °C and 60% relative humidity. Surface sterilization was performed using 1% sodium hypochlorite for 3 min, followed by three rinses in sterile distilled water. The seeds were imbibed on wet filter paper in Petri dishes at 4 °C in darkness for 3 days. Seed germination was induced in an LED growth chamber (Nippon Medical & Chemical Instruments, Osaka, Japan) under a 16 h light/8 h dark cycle at 23 °C. After 2–3 days, the seedlings were transplanted into plastic pots filled with 300 cm^3^ of soil (Sakata super mix A, Yokohama, Japan).

### 2.2. Aboveground Infection Assays

Three to four-week-old *B. distachyon* plants and 10-day-old barley plants were used to assess *R. solani* Japanese isolates’ virulence on aboveground tissues. A cylindrical mycelial plug (3 or 6 mm in diameter) was taken from the growing edge of a 3–5-day-old fungal culture and placed on individual leaves. Control plants received sterile PDA plugs of identical dimensions. Both inoculated and control plants were maintained in moisture chambers at 100% relative humidity and 23 °C, with a 16 h light/8 h dark photoperiod for 3 days.

### 2.3. Belowground Infection Assays

For belowground inoculation, the inoculum was prepared using a mixture of vermiculite (Uganda No 3, AICHI-mederu, Nishio, Japan) and wheat bran (Nisshin Seifun Welna, Tokyo, Japan) at a 2:1 (*w*/*w*) ratio. The mixture was placed in an autoclavable container, moistened with 300 mL of water, and autoclaved. Fifteen actively growing mycelial agar plugs (6–10 mm in diameter) from the edge of a 3–5-day-old culture were placed within the sterilized vermiculite–wheat bran mixture. The container was incubated at 23 °C under 100% relative humidity. After 7 days of fungal colonization, the inoculum was mixed with autoclaved soil (Sakata prime mix TKS-1, Yokohama, Japan) at a 1:4 ratio and transferred to plastic pots (~300 cm^3^). Each pot was watered with 30 mL of tap water, and the growth substrate was allowed to undergo fungal colonization for 2 additional days under the same conditions. Control pots were prepared using the same mixture without fungal inoculum. Seedlings (2–5-day-old, 2–3 cm roots for barley, and 5–6 cm roots for *B. disatchyon*) were carefully transplanted into the soil medium with or without fungal inoculum and incubated at 23 °C. A relative humidity of 100% was maintained for 2 days.

### 2.4. Phenotypic Measurements

For assessing infectivity on aboveground tissues, photographs were taken at 1, 2, and 3 days post-infection (dpi). The lesion area at 3 dpi was quantified using Leaf Doctor software (version 1.1; a quantitative assessment tool for plant diseases developed by Dr. Scot Nelson, University of Hawaii at Manoa) [39], and the measured lesion areas obtained for different genotypes were compared. To evaluate belowground virulence, three parameters were measured: number of dead seedlings, plant height, and fresh biomass. Plant height was measured from the base to the tip of the primary leaf. The fresh biomass of the shoots was determined by cutting plants at the base and immediately weighing them using a digital balance [40,41]. For each inoculated plant, the height and fresh biomass were compared to the mean values of non-inoculated control plants. Growth performance ratios were calculated by dividing each parameter value of inoculated plants by the mean value of the corresponding parameter in non-inoculated control plants [42]. The growth performance ratios were then analyzed alongside the mortality rates.

### 2.5. Phytohormone and NHP Treatment

The defense-inducing chemicals used in this study included sodium salicylate (Wako, Osaka, Japan), NHP (TargetMol, Boston, MA, USA), ethephon (an ethylene generator; Sigma-Aldrich, St Louis, MO, USA), and methyl jasmonate (Wako, Japan). The phytohormones were dissolved in dimethyl sulfoxide (DMSO) and diluted with distilled water to a final DMSO concentration of 0.1%. NHP was dissolved in water. Prior to pathogen inoculation, detached leaves or seedlings were either sprayed (~4 mL for every three plants, ensuring even coverage of the entire leaf surface) with the chemical solutions (100, 500, or 1000 µM) or subjected to soil drenching (30 mL per pot, with each pot containing three plants), followed by a 24 h incubation at 23 °C.

### 2.6. Fungal Biomass Quantification

Disease severity was assessed by quantifying fungal biomass using qPCR, following our previously reported protocol [15]. Genomic DNA was extracted from inoculated tissues using the Nucleospin Plant II Kit (Takara Bio, Shiga, Japan). qPCR was performed using Luna Universal qPCR Master Mix (NEB, Ipswich, MA, USA) on a LightCycler^®^ 96 system (Roche, IN, USA). PCR conditions consisted of a pre-incubation step at 95 °C for 60 s, followed by denaturation at 95 °C for 15 s and annealing and extension at 60 °C for 30 s, with 40 cycles. Melting was performed at 95 °C for 10 s, 65 °C for 60 s, and 97 °C for 1 s, and the data were analyzed using the equipped software. The primer sets specifically detecting *R. solani* AG-1 IA and AG-4 HG-I+II were used for each isolate, and *BdFIM* was used for normalization [14,43,44,45]. All primers used in this study are listed in Table S1.

### 2.7. Gene Expression Analysis

Total RNA was extracted from inoculated leaves using the ISOPIN Plant RNA Extraction Kit (NIPPON GENE, Tokyo, Japan). cDNA synthesis was performed with the PrimeScript™ RT Reagent Kit with gDNA Eraser (TaKaRa Bio, Kusatsu, Japan). Gene expression was quantified by quantitative reverse transcription polymerase chain reaction (qRT-PCR) using Luna Universal qPCR Master Mix (NEB, Ipswich, MA, USA) on a LightCycler 96 system (Roche, IN, USA). Expression levels were calculated using the 2^−ΔΔCt^ method, where ΔΔCt represents the difference in the ΔCt values between the inoculated samples and the non-inoculated controls at each time point. ΔCt was determined by subtracting the Ct value of the reference gene (to normalize the data) from the Ct value of the target gene for each sample. The reference genes used were *BdUbi4* (ubiquitin gene) for *B. distachyon* and *HvEF2* (elongation factor) for barley [46,47]. The primers and marker genes used in this study are listed in Tables S1 and S2.

### 2.8. Microscopy

The leaves of *B. distachyon* and barley were separately inoculated with *R. solani* AG-1 IA and AG-4 HG-I+II. Leaf samples (2–3 cm) containing the inoculation site, were collected in 2 mL tubes and fixed overnight in absolute ethanol until complete chlorophyll removal. Fungal mycelia were stained with Trypan Blue (Wako, Japan) in a mixture of equal parts by volume (*v*/*v*) of lactic acid, glycerol, phenol, and distilled water, and samples were stored in 30% glycerol. Samples were observed under a ZEISS Stemi 305 stereomicroscope, and photomicrographs were captured using an attached Axiocam 208 color camera (ZEISS, Oberkochen, Germany).

## 3. Results

### 3.1. Above- and Belowground Infectivity of R. solani Japanese Isolates on B. distachyon and Barley

We previously assessed the virulence of the Japanese field isolates of *R. solani* on the leaves of *B. distachyon* standard accession Bd21 [15]. Among the isolates tested, AG-1 IA (MAFF305230), AG-4 HG-I+II (MAFF305225), and AG-5 (MAFF305256) showed strong virulence, whereas AG-1 IA (MAFF305219) and AG-6 (MAFF305262) caused only mild symptoms (Table 1; see the note regarding the designation of the AG-4 isolate in Figure S1). In this study, we evaluated the infectivity of these isolates on the underground tissues of *B. distachyon* to investigate tissue-specific virulence. AG-4 HG-I+II and AG-6 caused strong and weak growth retardation of *B. distachyon* seedlings, respectively, while the other isolates were non-virulent on the roots (Table 1, Figure S1). Notably, AG-1 IA infected only the leaves, while AG-4 HG-I+II demonstrated strong virulence in both the leaves and roots of *B. distachyon*.

To assess host specificity, we further evaluated the infectivity of these *R. solani* isolates on the leaves and roots of the barley cultivar (cv.) ‘Golden Promise’, another monocot species. The isolates displayed similar pathogenicity patterns on barley leaves, with the exception that AG-2-1 II (MAFF305203) and AG-2-2 IIIB (MAFF305244) caused lower disease severity. Isolate AG-1 IA (MAFF305230), a known causal agent of rice sheath blight, and AG-4 HG-I+II caused the most severe symptoms with rapid disease progression. In the soil inoculation assays, the isolates showed pathogenicity patterns on barley roots similar to those observed for *B. distachyon.* Specifically, AG-4 HG-I+II caused strong growth retardation, while AG-6 caused weak growth retardation (Table 1, Figure S1).

In summary, the AG-1 IA isolate was highly virulent on the leaves of both plant species but did not infect root tissues. In contrast, the isolate AG-4 HG-I+II exhibited high virulence on both the leaves and roots of *B. distachyon* and barley.

### 3.2. Resistance Genotypes of B. distachyon and Barley Against R. solani

Previous studies have shown that *B. distachyon* accessions Bd3-1, Gaz-4, and Tek-3 exhibit relative resistance to leaf infection by the *R. solani* AG-1 IA isolate (MAFF305230) [15,48]. In this study, we investigated whether *B. distachyon* and barley possess genetic variation for resistance to AG-4 HG-I+II infection in both above- and belowground tissues.

**Figure 1:**
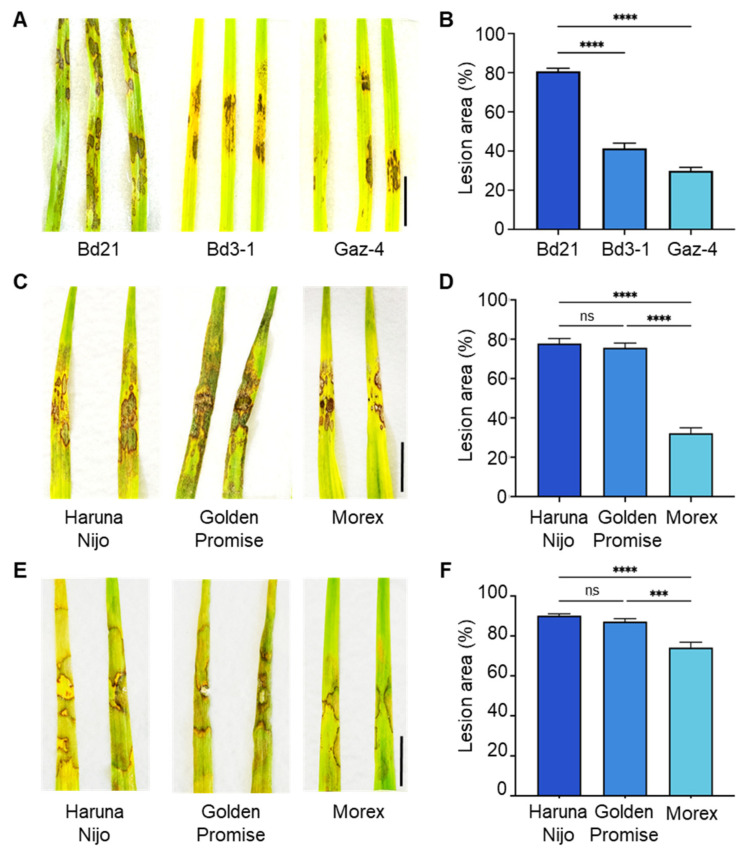
Differential leaf susceptibility to *R. solani* isolates among *B. distachyon* accessions and barley cultivars. (**A**,**B**) Disease symptoms (**A**) and quantified lesion areas (**B**) on detached leaves of *B. distachyon* accessions Bd21, Bd3-1, and Gaz-4 inoculated with *R. solani* AG-4 HG-I+II. (**C**,**D**) Disease symptoms (**C**) and quantified lesion areas (**D**) on detached leaves of barley cvs. Haruna Nijo, Golden Promise, and Morex inoculated with *R. solani* AG-4 HG-I+II. (**E**,**F**) Disease symptoms (**E**) and quantified lesion areas (**F**) on detached leaves of barley cultivars inoculated with *R. solani* AG-1 IA. Lesion areas are presented as mean percentages, i.e., the ratio of lesion area to total leaf area, ±SE (*n* = 6). Statistical significance was analyzed using one-way ANOVA followed by Tukey’s test (***, *p* < 0.001; ****, *p* < 0.0001; ns, not significant). Scale bars: 1 cm.

To evaluate resistance, we first compared lesion formation following AG-4 HG-I+II leaf inoculation among three *B. distachyon* accessions: Bd21, Bd3-1, and Gaz-4. Bd3-1 and Gaz-4 displayed milder symptoms compared to the susceptible accession Bd21 (Figure 1A,B). Similarly, we assessed AG-4 HG-I+II infectivity on the leaves of three barley cvs. ‘Haruna Nijo’, ‘Golden Promise’, and ‘Morex’. Barley cv. Morex showed relatively milder symptoms than Haruna Nijo and Golden Promise (Figure 1C,D). Additionally, we tested the susceptibility of these barley cultivars to AG-1 IA leaf infection. Once again, Morex displayed less severe symptoms compared to Haruna Nijo and Golden Promise (Figure 1E,F).

**Figure 2:**
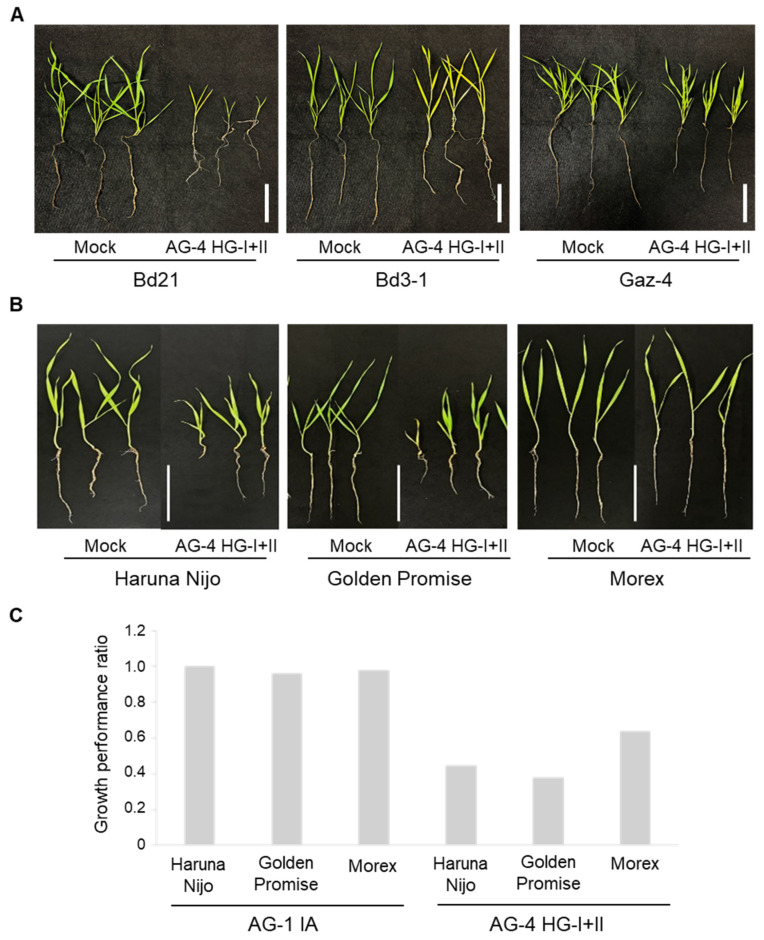
Root and shoot symptoms in *B. distachyon* accessions and barley cultivars grown in *R. solani* AG-4 HG-I+II-infested soil. (**A**) Representative images of *B. distachyon* accessions Bd21, Bd3-1, and Gaz-4 after 22 days of growth in soil infested with *R. solani* AG-4 HG-I+II. (**B**) Representative images of barley cvs. Haruna Nijo, Golden Promise, and Morex after 10 days of growth in soil infested with *R. solani* AG-4 HG-I+II. (**C**) Growth performance ratios (calculated by dividing each parameter value of inoculated plants by the mean value of the corresponding parameter in non-inoculated control plants) of three barley cultivars grown in soil infested with either *R. solani* AG-1 IA (left) or AG-4 HG-I+II (right). Scale bars: 5 cm.

We next evaluated root infection by AG-4 HG-I+II in three *B. distachyon* accessions and three barley cultivars. Among the *B. distachyon* accessions, Bd3-1 and Gaz-4 exhibited moderate resistance compared to the susceptible Bd21, showing better growth and lower mortality rates in the presence of the pathogen (Figure 2A, Table 2). Similarly, among barley cultivars, Morex displayed the lowest mortality rate and superior growth performance compared to Haruna Nijo and Golden Promise (Figure 2B,C, Table 2).

Additionally, we tested AG-1 IA root infection using the soil inoculation method on barley cvs. Haruna Nijo and Morex. The results confirmed that AG-1 IA is not virulent to these cultivars, consistent with its lack of virulence to Golden Promise under root inoculation conditions (Figure 2C, Table 1).

### 3.3. Comparison of Defense Responses in Leaves of Resistant B. distachyon and Barley Lines During Infection by R. solani AG-1 IA and AG-4 HG-I+II

Our study demonstrated that both *R. solani* AG-1 IA and AG-4 HG-I+II can infect the leaves of *B. distachyon* and barley. *B. distachyon* accession Bd21 and barley cvs. Haruna Nijo and Golden Promise were susceptible, whereas *B. distachyon* accessions Bd3-1 and Gaz-4 and barley cv. Morex were resistant to both AG-1 IA and AG-4 HG-I+II. Previous research showed that Bd3-1 and Gaz-4 activate SA-dependent immune responses immediately after infection with AG-1 IA [15].

**Figure 3:**
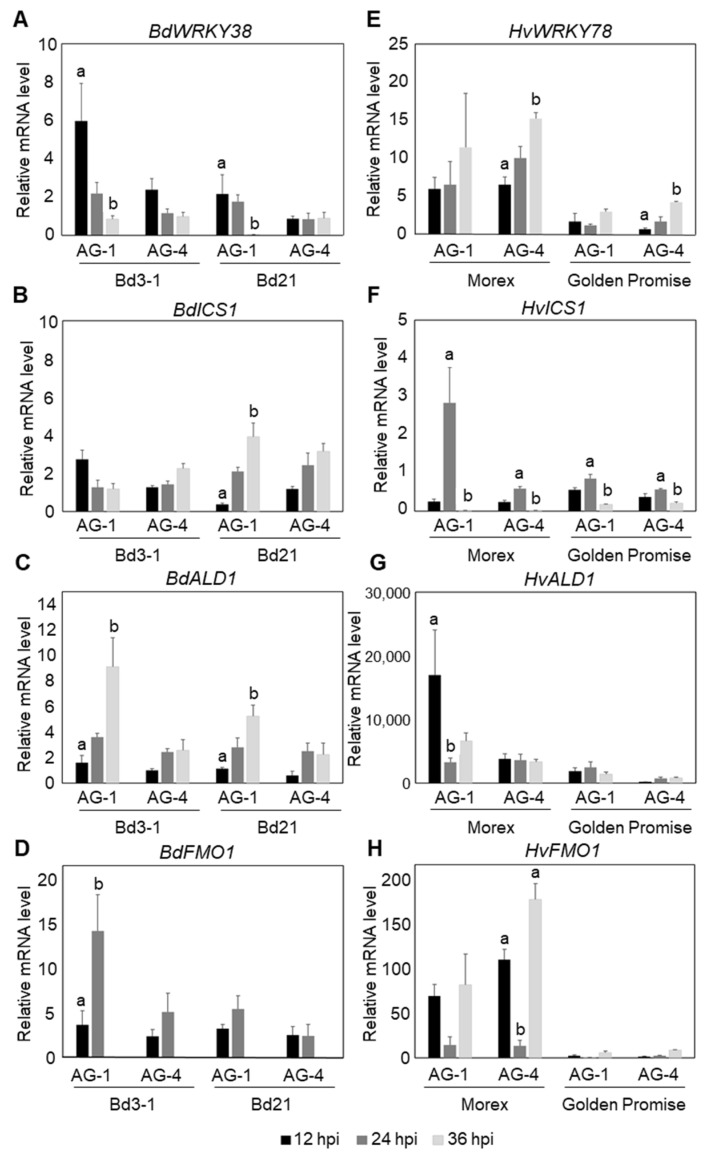
Temporal expression patterns of SA- and NHP-related defense genes in *B. distachyon* and barley leaves following *R. solani* AG-1 IA and AG-4 HG-I+II infection. (**A**–**D**) Expression analysis in resistant *B. distachyon* Bd3-1 and susceptible Bd21: (**A**) SA-responsive gene *BdWRKY38*, (**B**) putative SA biosynthesis gene *BdICS*, and putative NHP biosynthesis genes (**C**) *BdALD1* and (**D**) *BdFMO1*. (**E**–**H**) Expression analysis in resistant barley cv. Morex and susceptible cv. Golden Promise: (**E**) *HvWRKY78* (barley homolog of *BdWRKY38*), (**F**) putative SA biosynthesis gene *HvICS*, and putative NHP biosynthesis genes (**G**) *HvALD1* and (**H**) *HvFMO1*. Gene expression was quantified by qRT-PCR at 12, 24, and 36 hpi. Data are presented as means ± SEs from three independent biological replicates. Significant differences among time points for each isolate were determined using ANOVA followed by Tukey’s HSD. The letters above the bars indicate significant differences (*p* < 0.05). Comparisons are independent across isolates; therefore, the same letters in different isolates do not indicate comparability.

To determine whether similar responses occur against AG-4 HG-I+II, we used Bd3-1 and Morex as representative resistance lines for *B. distachyon* and barley, respectively. In *B. distachyon* Bd3-1, rapid and strong induction of *BdWRKY38* and *BdICS* (*Bradi4g28670*) was detected at 12 hpi with AG-1 IA, in agreement with our previous findings (Figure 3A,B). Additionally, potential NHP biosynthesis genes, *BdALD1* (*Bradi1g71530*) and *BdFMO1* (*Bradi1g72500*), which show sequence similarity to *A. thaliana AtADL1* and *AtFMO1*, whose functions have been characterized, were strongly upregulated at 36 and 24 hpi, respectively, during AG-1 IA infection (Figure 3C,D; a summary of the marker genes is given in Table S2). These results confirmed the induction of an SA-dependent response in Bd3-1 to AG-1 IA. In contrast, during infection by AG-4 HG-I+II, Bd3-1 exhibited only weak induction of these marker genes. Furthermore, the expression patterns of these genes differed distinctly from those observed during AG-1 IA infection.

In barley cv. Morex, a clear induction of *HvICS* (*HORVU5Hr1G057050*) was detected at 24 hpi during AG-1 IA infection (Figure 3F). Although this response was delayed by 12 h compared to the rapid induction observed in *B. distachyon* Bd3-1, the result suggests that a similar SA-dependent response may occur in the resistant barley cv. Morex. We selected *HvWRKY78* (*HORVU7Hr1G083270*) as the closest homolog to *BdWRKY38* based on amino acid sequence similarity. However, its expression patterns were similar during infection with both AG-1 IA and AG-4 HG-I+II (Figure 3E), suggesting that it may not specifically respond to SA. In contrast, *HvALD1* (*HORVU4Hr1G071300*), the closest homolog of *AtALD1*, was rapidly induced to AG-1 IA infection but showed no response to AG-4 HG-I+II in Morex (Figure 3G). Similarly, we identified *HvFMO1* (*HORVU4Hr1G077170*) as a homolog of *AtFMO1,* but its expression patterns were comparable during infection with AG-1 IA and AG-4 HG-I+II (Figure 3H).

These results suggest that although both Bd3-1 and Morex are resistant to AG-1 IA and AG-4 HG-I+II leaf inoculations, the underlying resistance mechanisms differ between these two plant species.

### 3.4. Effects of Exogenous SA and NHP Applications on Leaf Infection by R. solani AG-1 IA and AG-4 HG-I+II

**Figure 4:**
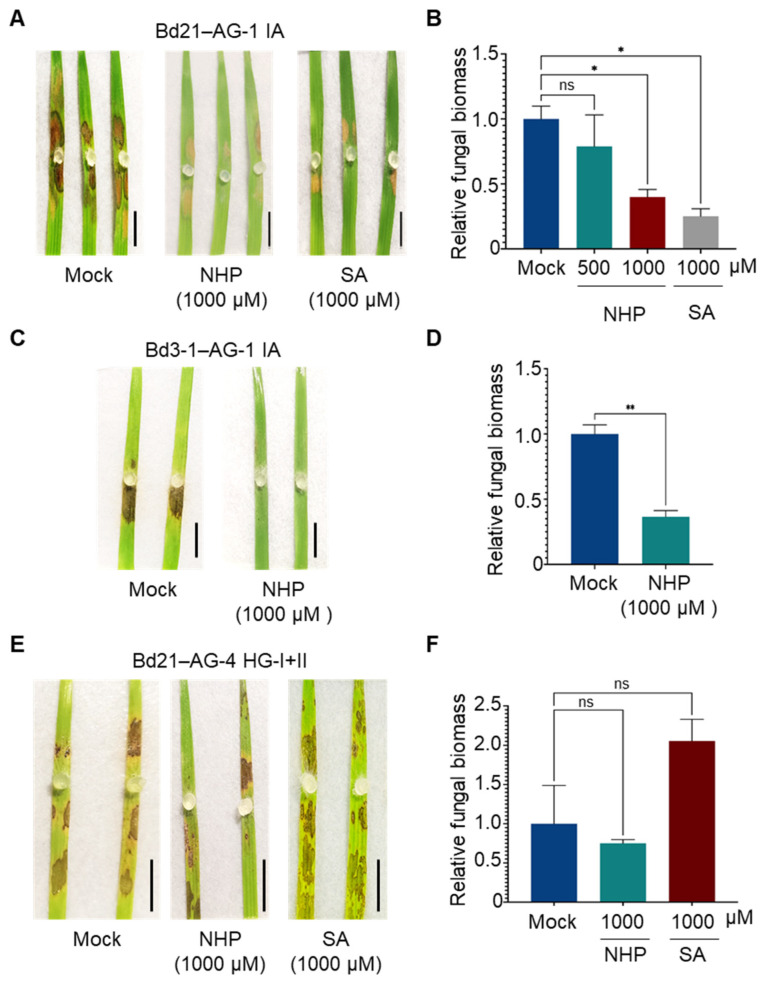
Differential effects of NHP and SA pretreatments on *R. solani* AG-1 IA and AG-4 HG-I+II infection in *B. distachyon* leaves. (**A**,**B**) Disease symptoms (**A**) and relative fungal biomass (**B**) in leaves of susceptible accession Bd21 pretreated with the indicated concentrations of NHP or SA before inoculation with *R. solani* AG-1 IA. (**C**,**D**) Disease symptoms (**C**) and relative fungal biomass (**D**) in leaves of resistant accession Bd3-1 pretreated with NHP before inoculation with *R. solani* AG-1 IA. (**E**,**F**) Disease symptoms (**E**) and relative fungal biomass (**F**) in leaves of susceptible accession Bd21 pretreated with NHP or SA before inoculation with *R. solani* AG-4 HG-I+II. Plants were pretreated with NHP, SA, or Mock (0.1% DMSO dissolved in water). Photographs were taken, and samples were collected for qPCR analysis at 48 hpi. Data are presented as means ± SEs (*n* = 3; a sampling unit is a randomly selected leaf representing an individual plant). Statistical significance was analyzed by one-way ANOVA followed by Dunnett’s test (*, *p* < 0.05; **, *p* < 0.001; ns, not significant). Scale bars: 1 cm.

To further confirm which defense responses are effective against *R. solani* AG-1 IA and AG-4 HG-I+II, we evaluated the effects of exogenously applied SA and NHP on the fungal infection. Intact *B. distachyon* plantlets were sprayed with SA or NHP at the indicated concentrations, and agar plugs of *R. solani* were inoculated onto leaves prepared from the treated plants. This approach yielded clearer effects of phytohormone pretreatments compared to treatments on detached leaves. Consistent with our previous findings, SA pretreatment reduced the symptoms caused by AG-1 IA in the susceptible *B. distachyon* accession Bd21 (Figure 4A,B). Similarly, NHP treatments suppressed AG-1 IA infection, with higher concentrations demonstrating greater efficacy (Figure 4A,B). The suppressive effects of SA and NHP on *R. solani* AG-1 IA were also observed when these compounds were applied by soil drenching (Figure S2). In the resistant accession Bd3-1, NHP pretreatment induced hyper-resistance to AG-1 IA (Figure 4C,D). In contrast, neither SA nor NHP pretreatment induced resistance to AG-4 HG-I+II in Bd21 (Figure 4E,F).

**Figure 5:**
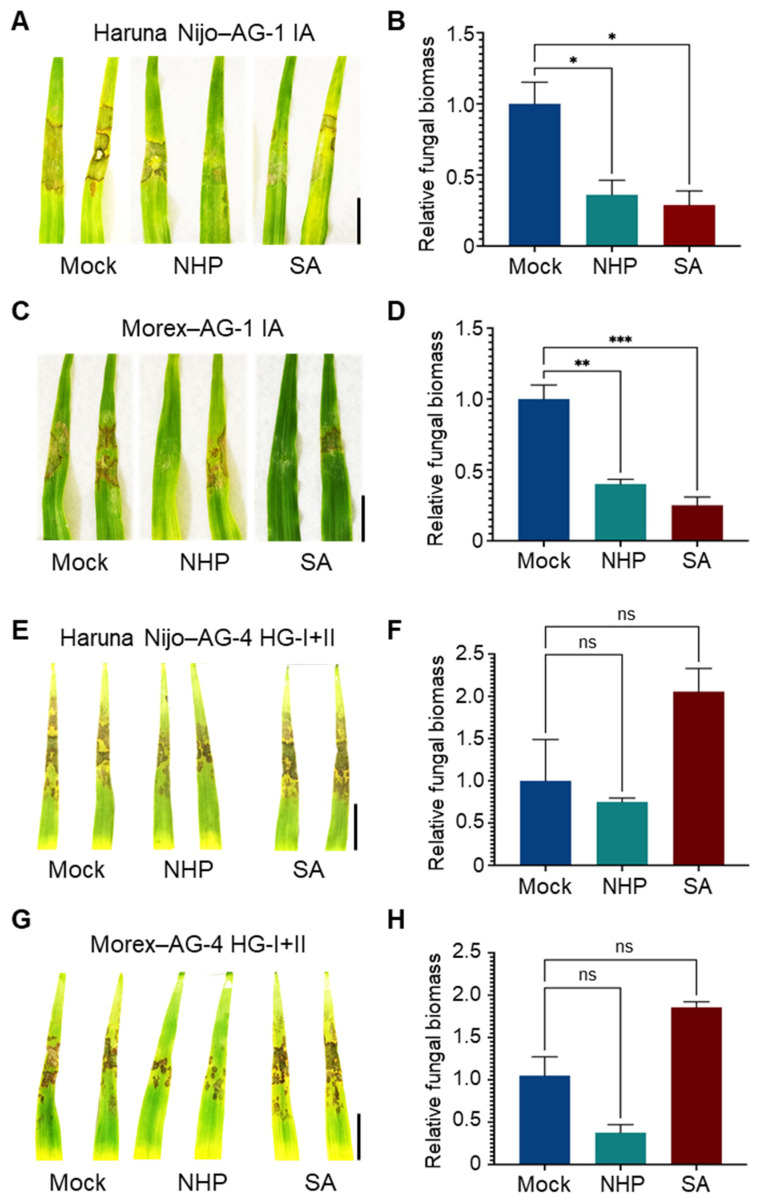
Differential effects of NHP and SA pretreatments on *R. solani* AG-1 IA and AG-4 HG-I+II infection in barley leaves. (**A**,**B**) Disease symptoms (**A**) and relative fungal biomass (**B**) in leaves of susceptible cv. Haruna Nijo pretreated with NHP or SA before inoculation of *R. solani* AG-1. (**C**,**D**) Disease symptoms (**C**) and relative fungal biomass (**D**) in leaves of resistant cv. Morex pretreated with NHP or SA before inoculation with *R. solani* AG-1 IA. (**E**,**F**) Disease symptoms (**E**) and relative fungal biomass (**F**) in leaves of susceptible cv. Haruna Nijo pretreated with NHP or SA before inoculation with *R. solani* AG-4 HG-I+II. (**G**,**H**) Disease symptoms (**G**) and relative fungal biomass (**H**) in leaves of resistant cv. Morex pretreated with NHP or SA before inoculation with *R. solani* AG-4 HG-I+II. Plants were pretreated with 1000 µM NHP or SA by foliar spray, with 0.1% DMSO dissolved in water as a mock control. Photographs were taken, and samples were collected for qPCR analysis at 48 hpi. Data are presented as means ± SEs (*n* = 3; a sampling unit is a randomly selected leaf representing an individual plant). Statistical significance was analyzed by one-way ANOVA followed by Dunnett’s test (*, *p* < 0.05; **, *p* < 0.01; ***, *p* < 0.001; ns, not significant). Scale bars: 3 cm.

Next, we evaluated the effects of these phytohormones in barley. Foliar pretreatment with SA or NHP reduced the symptoms caused by AG-1 IA in the susceptible barley cv. Haruna Nijo (Figure 5A), and resistance induction was further confirmed by a reduction in fungal biomass in the treated leaves (Figure 5B). Similar results were observed in the relatively resistant cv. Morex (Figure 5C,D). However, neither SA nor NHP induced resistance to AG-4 HG-I+II in either Haruna Nijo or Morex (Figure 5E–H). Instead, SA appeared to increase susceptibility to AG-4 HG-I+II in both cultivars, although this effect was not statistically significant. Additional experiments with varying SA concentrations (100, 500, and 1000 μM) revealed that higher concentrations of SA exacerbated disease severity caused by AG-4 HG-I+II (Figure S3).

Finally, we examined the effects of JA and ET on AG-4 HG-I+II infection in the leaves of *B. distachyon* and barley. However, neither phytohormone had any significant impact on infection outcomes, at least at the concentrations used (Figure S4).

### 3.5. Differences in Infection Behavior Between R. solani AG-1 IA and AG-4 HG-I+II

The differences in induced defense responses against *R. solani* AG-1 IA and AG-4 HG-I+II in both *B. distachyon* and barley suggest that the infection mechanisms of these isolates may differ, despite these isolates belonging to the same genus and species. To explore this further, we conducted microscopic observations of mycelial growth and infection cushion formation on both plant species. Infected leaves were observed at 20 hpi in *B. distachyon* and 32 hpi in barley.

**Figure 6:**
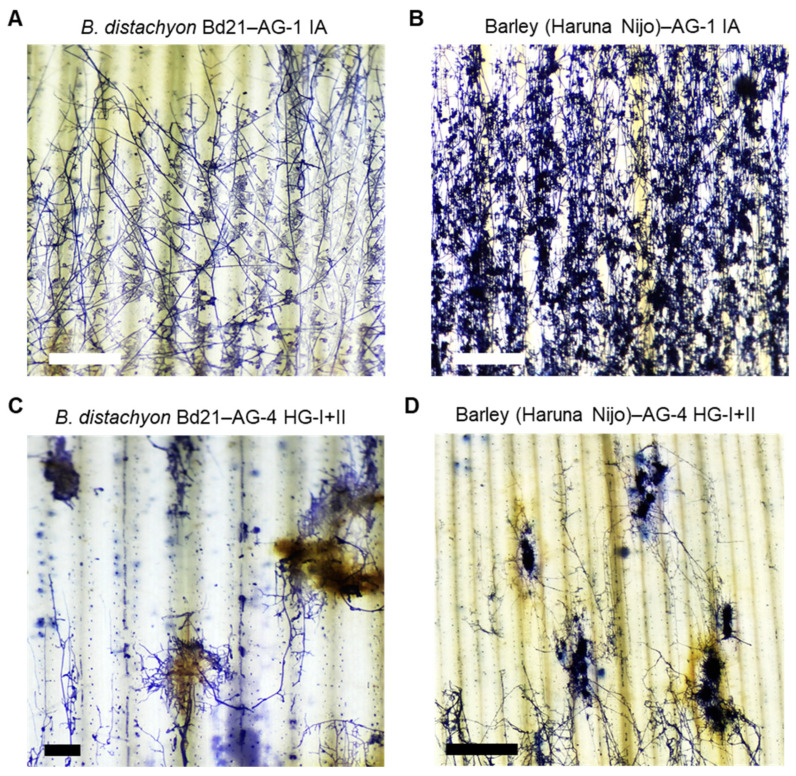
Hyphal growth of *R. solani* AG-1 IA and AG-4 HG-I+II on infected *B. distachyon* and barley leaves. (**A**,**B**) Hyphae of *R. solani* AG-1 IA on the leaf surface of susceptible *B. distachyon* accession Bd21 (**A**) and susceptible barley cv. Haruna Nijo (**B**). (**C**,**D**) Hyphae of *R. solani* AG-4 HG-I+II on the leaf surface of susceptible *B. distachyon* accession Bd21 (**C**) and susceptible barley cv. Haruna Nijo (**D**). Leaves were sampled at 20 hpi for *B. distachyon* and 36 hpi for barley and then stained with trypan blue. Scale bars: 1 cm.

In *B. distachyon* Bd21 infected with AG-1 IA, fungal hyphae spread evenly across the leaves, and massive infection cushions were detected throughout the infected area, consistent with previous reports (Figure 6A) [15,48]. Similarly, in barley cv. Haruna Nijo, AG-1 IA formed a widely dispersed mycelial network with abundant infection cushions covering the entire leaf surface at this time point (Figure 6B).

In contrast, AG-4 HG-I+II displayed a distinct infection pattern in both *B distachyon* and barley. Fungal mycelia grew in aggregated clusters, forming thick, encircling masses around specific infection sites (Figure 6C,D). Connections between these mycelial aggregates were sparse, resulting in a dotted hyphal distribution compared to the extensive mycelial networks and infection cushion connections observed in AG1-IA infections. The leaf tissue surrounding these hyphal aggregates of AG-4 HG-I+II exhibited necrosis with pronounced brownish pigmentation in both plant species (Figure 6C,D and Figure S4).

## 4. Discussion

In this study, we evaluated the infectivity of selected Japanese field isolates of *R. solani* on two cereal plants, *B. distachyon* and barley. Among the tested isolates, AG-1 IA (MAFF305230) and AG-4 HG-I+II (MAFF305225) were highly virulent on the aboveground tissues of both plant species, but only AG-4 HG-I+II exhibited virulence on the belowground tissues.

*R. solani* AG-1 IA (MAFF305230), originally isolated from rice sheath blight in Fukuoka, Japan, is representative of isolates frequently associated with this disease globally [49]. Historically, *R. solani* AG-1 and AG-2 have been recognized for their broad host ranges [50]. Previous studies showed that AG-1 IA is highly virulent on the aboveground tissues of *B. distachyon* [15] and on both the above- and belowground tissues of *A. thaliana* [16]. When grown in identical soil (growth substrate) conditions, AG-1 IA infected the roots of *A. thaliana* but not *B. distachyon*, suggesting tissue-specific virulence patterns.

*R. solani* AG-4 HG-I+II (MAFF305225), isolated from cauliflower plants in Fukushima, is a recently classified isolate [14]. While *R. solani* AG-8 has historically been the primary AG associated with Rhizoctonia root disease in cereals in the USA and Australia, AG-4 and AG-6 have been identified as pathogens in regions including the USA, Turkey, Azerbaijan, South Africa, and Tanzania. AG-4 is known to cause seedling blight in rice [51] and foliar blights in barley [52,53,54]. Additionally, AG-4 affects a wide range of dicot crops [54]. A Japanese AG-4 isolate from peanuts was shown to infect wheat under controlled conditions [55], and AG-4 HGI3, isolated from buckwheat, exhibited a broad host range [38].

Understanding the molecular mechanisms of *R. solani* virulence is essential for developing effective crop protection strategies. However, progress has been limited by the challenges associated with genetic engineering in this pathogen. To overcome this limitation, we focused on exploring host resistance mechanisms. Kidd et al. (2021) demonstrated the role of defense barriers in Arabidopsis nonhost resistance against leaf and root infections by AG-8 [34]. Building on this approach, our first strategy involved leveraging genetic variation in host resistance. Previously, we identified *B. distachyon* accessions Bd3-1 and Gaz-4 as resistant to AG-1 IA leaf inoculation [15]. In this study, we observed AG-1 IA virulence on barley leaves, with cv. Morex showing moderate resistance compared to the more susceptible cvs. Haruna Nijo and Golden Promise. Similarly, *B. distachyon* accessions Bd3-1 and Gaz-4 demonstrated resistance to AG-4 HG-I+II leaf infection, similar to their resistance to AG-1 IA. Barley cv. Morex also exhibited resistance to AG-4 HG-I+II leaf infection. When evaluating root infection by AG-4 HG-I+II, *B. distachyon* Bd3-1 and Gaz-4, along with barley cv. Morex, displayed enhanced resistance compared to other accessions and cultivars. Previous studies have also highlighted genetic variation in *B. distachyon* resistance to AG-8 root infection, with accessions Bd3-1, Koz-3, and Bd30-1 showing relatively higher resistance than Bd21 [40]. These findings suggest that plants employ diverse strategies to combat *R. solani*. The identification and pyramiding of such resistance traits could pave the way for effective, durable strategies to control this pathogen and protect valuable crops.

The second strategy focused on defense responses and phytohormone-induced resistance. Among the plants tested against *R. solani* isolates AG-1 IA and AG-4 HG-I+II, *B. distachyon* accessions Bd3-1 and Gaz-4, and barley cv. Morex consistently showed resistance. Initially, we hypothesized that these plants might use similar resistance mechanisms against both isolates. However, the expression patterns of defense-related marker genes during infection revealed distinct responses to each pathogen isolate. In Bd3-1 and Morex, genes associated with SA and NHP biosynthesis were induced earlier in response to AG-1 IA than to AG-4 HG-I+II. This suggests that SA- and NHP-related immunity specifically targets AG-1 IA. Chemical pretreatment experiments supported this hypothesis: pretreatment with either SA or NHP effectively suppressed AG-1 IA infection in both resistant and susceptible *B. distachyon* accessions and barley cultivars but had no impact on AG-4 HG-I+II. While previous studies demonstrated that SA pretreatment induced resistance to AG-1 IA in *B. distachyon* and rice, we now show that NHP similarly induces defense against this isolate in both *B. distachyon* and barley. NHP, a recently identified mobile signal for SAR, functions in concert with SA to defend against biotrophic pathogens. SA accumulates in locally infected tissues, triggering NHP synthesis, which then travels to systemic tissues and amplifies SA production and defense responses [56]. Enhanced NHP biosynthesis has been linked to resistance against biotrophic and hemibiotrophic pathogens in monocots, including *B. distachyon* and barley [57]. Similarly, Pip pretreatment reduced infections by hemibiotrophic pathogens such as *Xanthomonas translucens* pv. *cerealis* and *Blumeria graminis* f. sp. *hordei* in barley [32]. Although Pip pretreatment did not significantly enhance resistance to the necrotrophic pathogen *Pyrenophora teres*, it modestly reduced lesion size without negative effects [32]. We previously demonstrated that SA-related immunity plays a significant role in Bd3-1′s resistance to AG-1 IA through BdWRKY38, a key regulator of SA signaling [48]. The effectiveness of NHP pretreatment against AG-1 IA further supports the existence of a biotrophic interaction phase during its infection of *B. distachyon* and barley leaves. Such biotrophic infection and host recognition of pathogens could be mediated by the effector proteins released by *R. solani*.

In contrast, neither SA nor NHP pretreatment was effective against AG-4 HG-I+II leaf infections in *B. distachyon* or barley, suggesting different infection mechanisms between the two isolates. These results imply that AG-4 HG-I+II operates as a purely necrotrophic pathogen or has a very brief biotrophic phase compared to AG-1 IA, which exhibits a hemibiotrophic lifestyle. Consistently, neither *B. distachyon* Bd3-1 nor barley cv. Morex induced SA- or NHP-biosynthesis genes during AG-4 HG-I+II infection. Although both Bd3-1 and Morex are resistant to AG-1 IA, their resistance mechanisms against AG-1 IA and AG-4 HG-I+II appear to differ. This is further supported by the observation that Bd3-1′s resistance to AG-1 IA was compromised by the expression of the bacterial *NahG* gene, which encodes SA hydroxylase and degrades SA [15,48]. This result suggests that SA is necessary for resistance in Bd3-1 to *R. solani* AG-1 IA. Understanding these diverse defense mechanisms provides valuable insights into nonhost resistance and may inform the development of resistant crop varieties.

Consistent with these findings, *R. solani* AG-1 IA and AG-4 HG-I+II exhibited distinct infection behaviors. AG-1 IA infected leaves by forming an extensive mycelial network with abundant infection cushions, whereas AG-4 HG-I+II produced localized, compact mycelial masses in dispersed areas. Our results suggest that the *R. solani* species complex contains at least two types of pathogens with different styles of infection. Infection cushion formation, a hallmark of the penetration stage, has been well-documented in sheath blight disease [58,59,60,61]. Studies have reported a correlation between disease resistance and the number and distribution of infection cushions [36,60]. We previously demonstrated that SA pretreatment prevented AG-1 IA progression to the infection cushion-forming stage on *B. distachyon,* and resistant *B. distachyon* accessions blocked infection cushion formation entirely [15,48]. This suggests that *R. solani* isolates utilizing infection cushions may adopt a biotrophic phase before transitioning to necrotrophy, marked by extensive tissue penetration through these specialized structures. In contrast, no phytohormone pretreatments tested were effective against AG-4 HG-I+II in *B. distachyon* or barley. This is notable because JA and ET pretreatments typically induce resistance against some necrotrophic pathogens [62]. Consistent with this, previous studies have shown that SA application can increase susceptibility to necrotrophic pathogens, such as *Alternaria brassicicola,* by suppressing JA-related defense mechanisms [63]. Similarly, SA pretreatment enhanced tomato susceptibility to *Botrytis cinerea* [64], although in *A. thaliana*, both SA and JA are required for resistance against *B. cinerea* [65]. We previously reported that no defense-related phytohormone pretreatments conferred resistance to either AG-1 IA or AG-4 HG-I+II in *A. thaliana* [16]. These findings suggest that the necrotrophic lifestyle of AG-4 HG-I+II in *B. distachyon* and barley, as well as that of both AG-1 IA and AG-4 HG-I+II in Arabidopsis, may parallel the behavior of well-studied necrotrophic pathogens like *B. cinerea* and A. *brassicicola.*

We hypothesize that AG-4 HG-I+II employs secreted CAZymes and/or toxins to kill host cells prior to invasion. This strategy, which may limit direct contact with host defenses, renders chemically or physically induced barriers by phytohormones ineffective. Observations showed that AG-4 HG-I+II induced necrosis beneath hyphal masses more rapidly than AG-1 IA. The resulting dead host tissue, characterized by brownish pigment accumulation, further supports its necrotrophic lifestyle. A recent genomic comparison between AG-1 IA and AG-4 HGI3 (isolated from buckwheat) revealed that AG-4 HGI3 possesses significantly more CAZymes, including cellulases, ligninases, hemicellulases, and pectinases [38]. These CAZymes likely enable its necrotrophic lifestyle. He et al. (2023) also demonstrated that JA-mediated resistance contributes to the AG-4 HGI 3 isolate in Tartary buckwheat, suggesting another different type of virulence mechanism in the *R. solani* species complex [38]. In conclusion, the differential responses to SA pretreatment between AG-1 IA and AG-4 HG-I+II in *B. distachyon* and barley leaves reflect AG-1 IA’s hemibiotrophic nature versus AG-4 HG-I+II’s necrotrophic strategy. Further studies focusing on host responses and microscopic analysis of hyphal infection behavior may uncover additional lifestyle adaptations within the *R. solani* species complex, which have evolved to target diverse hosts.

## Figures and Tables

**Table 1:** The infectivity of Japanese field isolates of *R. solani* on the above- and belowground tissues of *B. distachyon* and barley.

*R. solani* Isolates				Infectivity ^3^			
				*B. distachyon*		Barley	
Anastomosis Group (AG)	MAFF Number ^1^	Name	Source	Leaves ^4^	Roots	Leaves	Roots
AG-1 IA	305230	C-325	Rice	++	−	++	−
AG-1 IA	305219	C-54	Rice	+	−	+	−
AG-2-1 II	305203	6	Barley	−	−	+	−
AG-2-2 IIIB	305244	C-329	Rice	−	−	+	−
AG-3 IV	305250	C-564	Potato	−	−	−	−
AG-4 HG-I+II ^2^	305225	BO-3	Cauliflower	++	++	++	++
AG-5	305256	SH-30	Soil	−	−	−	−
AG-6	305262	UB-7-1-A	Soil	−	+	−	+

^1^ MAFF numbers are descriptors assigned by the National Agriculture and Food Research Organization (NARO) in Japan for microorganism genetic resources. The AGs and subgroups of the *R. solani* isolates used were accurately characterized by Misawa and Kukrose (2019) [14]. ^2^ We previously noted this isolate as AG-4 IIIA in Abdelghany et al. (2020) [16], but it was re-designated as AG-4 HG-I+II by Misawa and Kurose (2019) [14]. ^3^ (++)—severe symptoms, (+)—moderate symptoms, (−)—no symptoms. The severity scales were assigned based on the percentage of leaf lesion area for aboveground infections and the growth retardation ratio for belowground infections (Figure S1). ^4^ Data were retrieved from Kouzai et al. (2018) [15].

**Table 2:** Phenotypic responses of different genotypes of *B. distachyon* (22 dpi) and barley (10 dpi) to belowground infection of *R. solani* AG-4 HG-I+II.

Plant Species	Genotype	Treatment	Height (cm) ^1^	Height Ratio ^2^	Fresh Weight (mg) ^1^	Fresh Weight Ratio ^2^	Root Development ^3^	Mortality Rate (%)
*Brachypodium distachyon*	Bd21	Control	7.57 ± 0.10		14.35 ± 0.15			
		*R. solani*	2.83 ± 0.24 *	0.37 ^a^	5.72 ± 0.64 *	0.39 ^a^	+	50
	Bd3-1	Control	7.87 ± 0.12		14.15 ± 0.30			
		*R. solani*	5.13 ± 0.25 *	0.65 ^ab^	7.97 ± 0.59 *	0.56 ^ab^	++	25
	Gaz4	Control	8.13 ± 0.10		15.44 ± 0.19			
		*R. solani*	5.96 ± 0.18 *	0.73 ^b^	10.45 ± 0.34 *	0.67 ^b^	++	18.75
Barley	Haruna Nijo	Control	19.79 ± 1.37		683.35 ± 20.23			
		*R. solani*	8.92 ± 0.42 *	0.45 ^a^	259.83 ± 65.91 *	0.38 ^a^	+	20
	Golden Promise	Control	11.94 ± 0.83		346.34 ± 22.51			
		*R. solani*	3.76 ± 0.18 *	0.31 ^a^	169.01 ± 46.28 *	0.48 ^a^	+	30
	Morex	Control	20.49 ± 1.27		543.89 ±18.85			
		*R. solani*	13.21 ± 0.67 *	0.64 ^b^	482.28 ± 40.94 *	0.88 ^b^	++	0

^1^ Statistical differences for height and fresh weight between inoculated and non-inoculated experiments were analyzed using Student’s *t*-tests (*n* = 10, *, *p* < 0.05). ^2^ Statistical differences for the height ratio and fresh weight ratio among accessions and cultivars were analyzed using the Kruskal–Wallis rank sum test. The ratio obtained for the inoculated plant was divided by the corresponding value obtained for the control plant. ^3^ (++)—Good root development, (+)—poor root development (Figure S1).

## Data Availability

The data are contained within this article or Supplementary Materials.

## Supplementary Materials

The following supporting information can be downloaded at https://www.mdpi.com/article/10.3390/life15020235/s1: Figure S1. Criteria for the disease severity assay caused by *R. solani* isolates in the leaf and root inoculation in Table 1 and Table 2; Figure S2: Root-applied salicylic acid and *N*-hydroxypipecolic acid affect *R. solani* AG-1 IA infection on *B. distachyon* leaves; Figure S3: Salicylic acid pretreatment increases susceptibility to *R. solani* AG-4 HGI+II in resistant *B. distachyon* and barley leaves; Figure S4: Jasmonic acid and ethylene pretreatments do not affect *R. solani* AG-4 HG-I+II infection in susceptible *B. distachyon* and barley leaves; Figure S5: Necrotrophic lesions under *R. solani* AG-4 HG-I+II mycelial masses on leaves of susceptible barley cv. Haruna Nijo; Table S1: Primers used in this study; Table S2: Marker genes analyzed in this study.
