# Supplementary material for: Distinct Infection Mechanisms of Rhizoctonia solani AG-1 IA and AG-4 HG-I+II in Brachypodium distachyon and Barley
Source: Life (Basel). 2025 Feb 5;15(2):235. doi: 10.3390/life15020235 (PMC11856681; doi:10.3390/life15020235)
Supplement: Supplementary file 1 [file life-15-00235-s001.zip › life-3440661-supplementary.pdf]

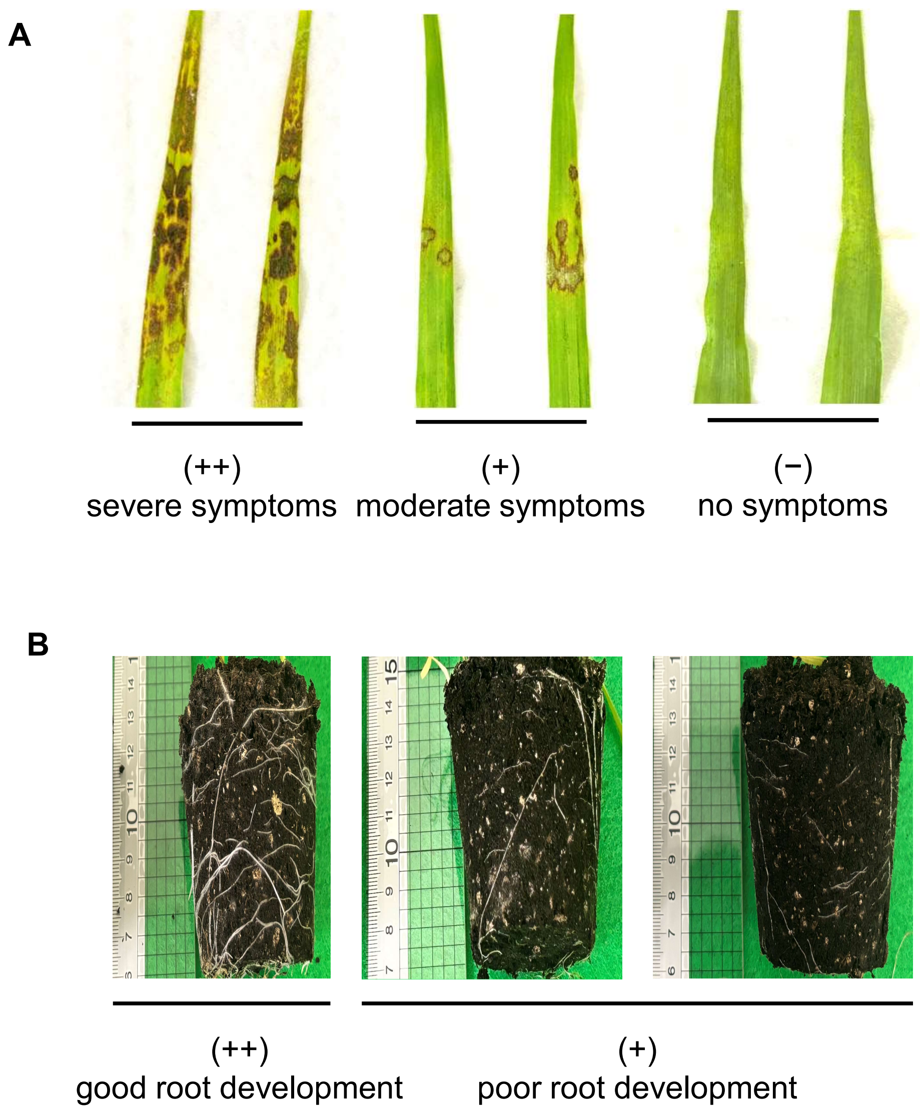

Figure S1. Criteria for the disease severity assay caused by *R. solani* isolates in the leaf and root inoculation in Tables 1 and 2. (A) Barley leaves inoculated with *R. solani* isolates AG-4 HG-I+II (severe symptoms), AG-2-2 IIIB (moderate symptoms), and AG-5 (no symptoms). (B) Root development in barley cultivars Morex (left, good root development), Haruna Nijo (middle, poor root development), and Golden Promise (right, poor root development) infected with *R. solani* isolate AG-4 HG-I+II. Photographs were taken 10 days after inoculation.

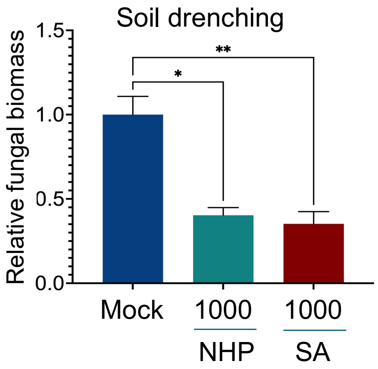

Figure S2. Root-applied salicylic acid and *N*-hydroxypipecolic acid affect *R. solani* AG-1 IA infection on *B. distachyon* leaves. Salicylic acid and *N*-hydroxypipecolic acid were applied at the indicated concentrations via soil drenching, with 0.1% DMSO serving as a mock control. After 24 hours, detached leaves were inoculated with AG-1 IA. Relative fungal biomass was quantified by qPCR from leaves harvested at 48 hpi. Data are presented as mean ± SE (n = 3). Statistical significance was determined by one-way ANOVA followed by Dunnett's test (*, $P < 0.05$; **, $P < 0.001$; ns, not significant).

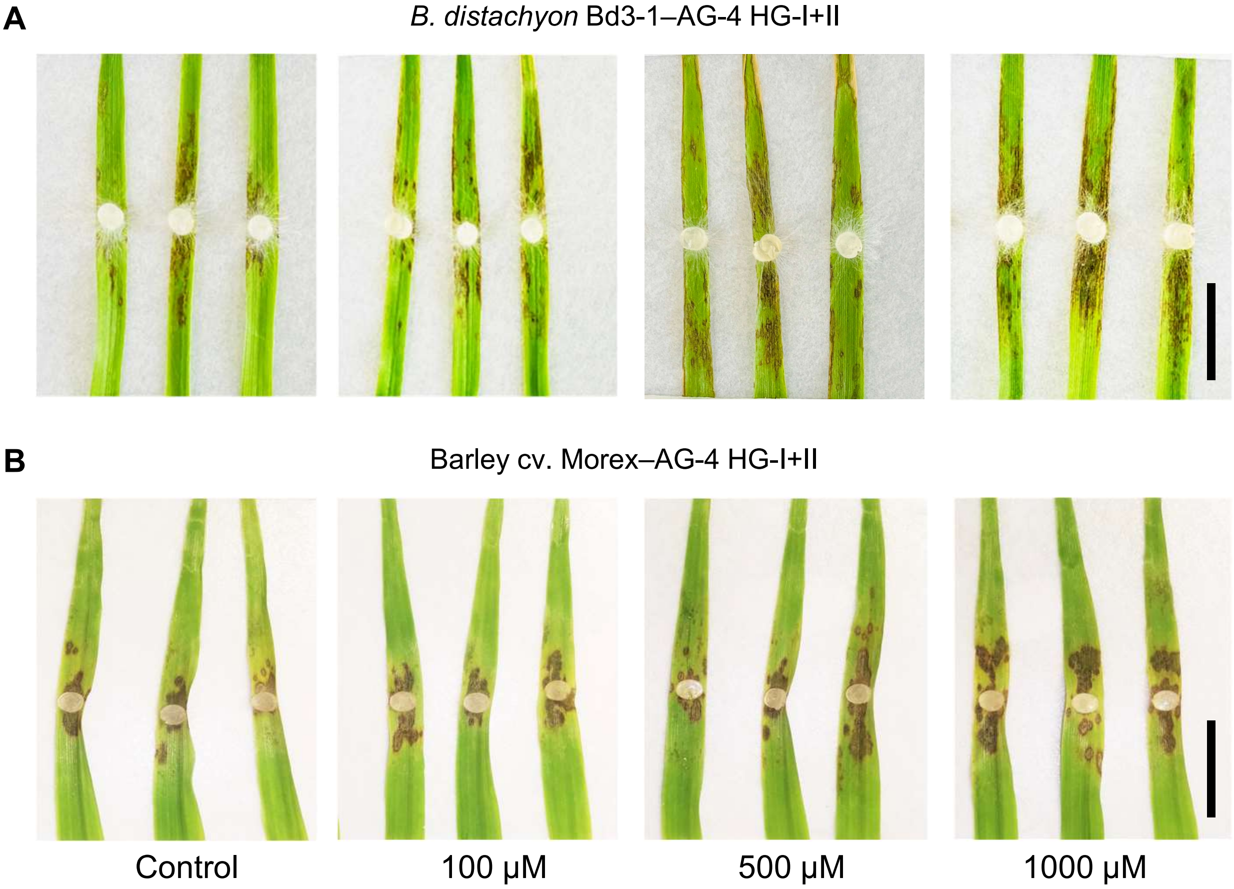

Figure S3. Salicylic acid pretreatment increases susceptibility to *R. solani* AG-4 HGI+II in resistant *B. distachyon* and barley leaves. Leaves from resistant *B. distachyon* Bd3-1 **(A)** and barley cv. Morex **(B)** were pretreated with varying concentrations of salicylic acid or 0.1% DMSO (mock control) for 24 h before indication with *R. solani* AG-4 HGI+II. Photographs were taken at 48 hpi. Scale bars: 2.5 cm **(A)** and 3.0 cm **(B)**.

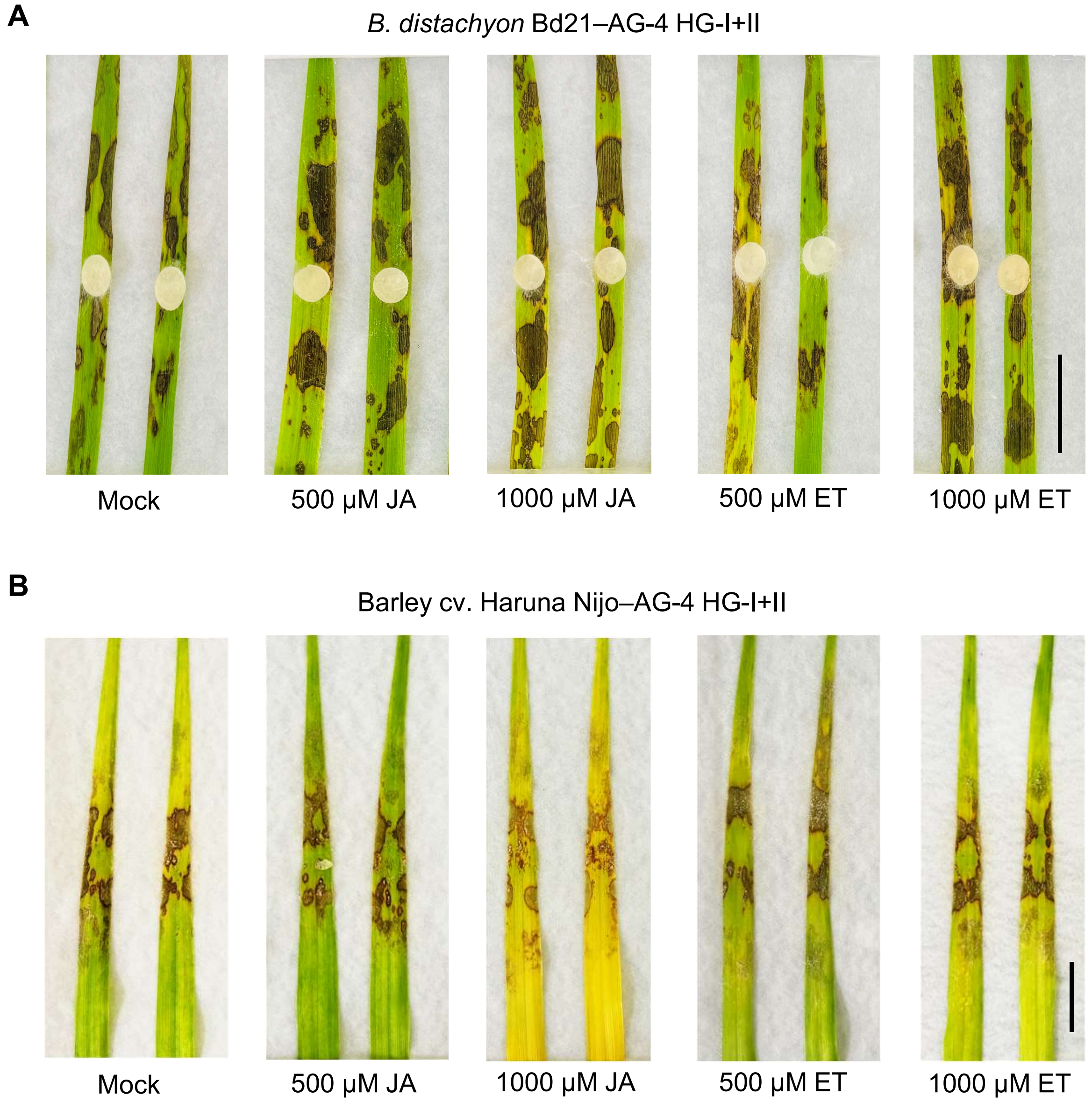

Figure S4. Jasmonic acid and ethylene pretreatments do not affect *R. solani* AG-4 HG-I+II infection in susceptible *B. distachyon* and barley leaves. Susceptible *B. distachyon* accession Bd21 **(A)** and barley cv. Haruna Nijo **(B)** were sprayed with phytohormones at the indicated concentrations, with 0.1% DMSO serving as a mock control. Leaves were subsequently inoculated with *R. solani* AG-4 HG-I+II. Photographs were taken at 48 hpi. Scale bars: 2.0 cm **(A)** and 2.5 cm **(B)**.

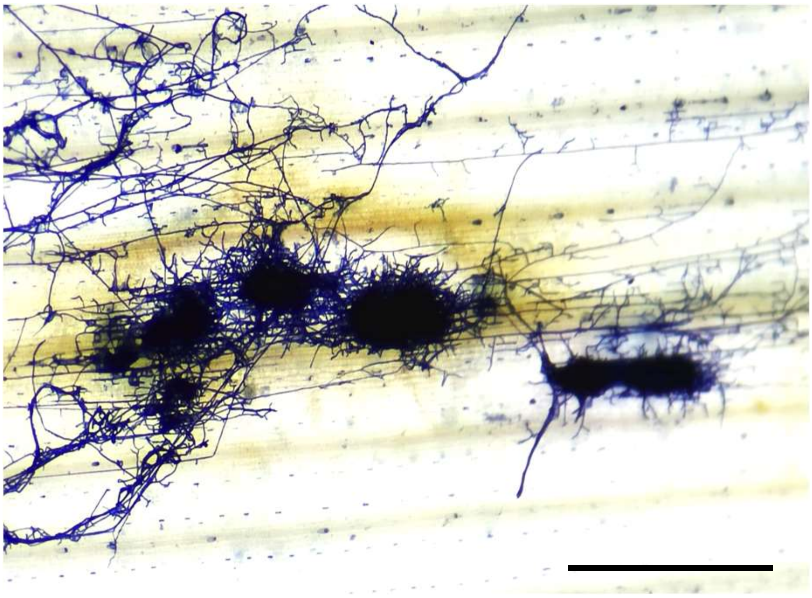

Figure S5. Necrotrophic lesions under *R. solani* AG-4 HG-I+II mycelial masses on leaves of susceptible barley cv. Haruna Nijo. Leaves were sampled at 36 hpi and stained with trypan blue to visualize hyphae. Scale bar: 200 μm.

Table S1. Primers used in this study

Target	Primer name	Sequence	Reference
AG-1 IA	Rs-1F	GCCTTTTCTACCTTAATTTGGCAG	Sayler and Yang, 2007
	Rs-2R	GTGTGTAAATTAAGTAGACAGCAAATG	
AG-4 HG-I+II	AG-4 HG-I+II-F	TGTGCACCTGTGAGACAGATGTT	Budge et al. 2009
	AG-4 HG-I+II-R	TGTGTTACATCCATTACATCCGTT	
BdFIM (*Bradi2g13800*)	BdFIM-F	CCTCACACGGATTTCGAGAGA	Zhu et al., 2014
	BdFIM-R	GGACAACCCATTTCTGCGA	
BdUbi4 (*Bradi3g04730*)	BdUbi4-F	TGACACCATCGACAACGTGA	Chambers et al., 2012
	BdUbi4-R	GAGGGTGGACTCCTTCTGGA	
BdICS (*Bradi4g28670*)	BdICS-F	AGGGAAAACAAGGGCTGATG	This study
	BdICS-R	TTGCTGGGATGGACAACAAC	
BdFMO1 (*Bradi1g72500*)	BdFMO1-F	TCTCCCAGCTCTTCTACGAAAG	This study
	BdFMO1-R	CGACTCGATGAATTTGGACACC	
BdALD1 (*Bradi1g71530*)	BdALD1-F	TTTGAGTCGTTGGGGAAGGAG	This study
	BdALD1-R	CACCGTGATAACATGCGTCTTC	
BdWRKY38 (*BdWRKY45L1*) (*Bradi2g30695*)	BdWRKY38-F	GCACGCACAAGTACGACCAG	Kouzai et al., 2018<br>Kouzai et al., 2020
	BdWRKY38-R	TGCACGCCGATGTATGTCAC	
HvEF2 (*AK250157*)	HvEF-2-F	AACTGGCATGAAGGTCCGTA	Gines et al., 2018
	HvEF-2-R	GGCAGGCATCAACTTCCTTC	
HvICS (*HORVU5Hr1G057050*)	HvICS-F	TGACGCTCCTGCATTTGTTG	This study
	HvICS-R	ATCATCGGCCCTTGTTTTCC	
HvFMO1 (*HORVU4Hr1G077170*)	HvFMO1-F	TGCATCTCCACCTACAGCATC	This study
	HvFMO1-R	TGTTGTTGTATGGCGCGAAG	
HvALD1 (*HORVU4Hr1G071300*)	HvALD1-F	GCTTCGCAATCCAAACATGG	This study
	HvALD1-R	ACTTCTTCTGGTGTGCTTCG	
HvWRKY78 (*HORVU7Hr1G083270*)	HvWRKY78-F	TACACAACTCCAAGCACTCGAG	This study
	HvWRKY78-R	TGTAGGTGACCCTGAACATGC	

Table S2. Marker genes analyzed in this study

Gene name	*B. distachyon* ID[1]	Barley ID[1]	Role
WRKY38	*Bradi2g30695*	NA[2]	Positively regulates SA signaling pathway
WRKY78	NA[2]	HORVU7Hr1G083270	Closest homologous to *BdWRKY38*
ICS	*Bradi4g28670*	HORVU5Hr1G057050	Possible SA biosynthesis
ALD1	*Bradi1g71530*	HORVU4Hr1G071300	NHP biosynthesis, converts L-lysine to dehydropipecolic acid
FMO1	*Bradi1g72500*	HORVU4Hr1G077170	NHP biosynthesis, converts Pip to NHP

[1]ID is based on Phytozome13, Plant Genetic Resource data base
[2]Not applicable
SA: Salicylic acid
NHP: *N*-hydroxy pipecolic acid
